# Bevacizumab-induced intestinal perforation in a patient with inoperable breast cancer: a case report and review of the literature

**DOI:** 10.1186/s13256-018-1619-x

**Published:** 2018-03-27

**Authors:** Yusuke Fujii, Noriyuki Hirahara, Syunsuke Kaji, Takahito Taniura, Ryoji Hyakudomi, Tetsu Yamamoto, Yoshitsugu Tajima

**Affiliations:** Department of Digestive and General Surgery, Shimane University, Faculty of Medicine, 89-1, Enyacho, Izumo-shi, Shimane-prefecture 693-8501 Japan

**Keywords:** Bevacizumab, Breast cancer, Gastrointestinal perforation

## Abstract

**Background:**

Gastrointestinal perforation is known as a serious adverse event, but, for breast cancer, there are very few reports of gastrointestinal perforation. This report highlights gastrointestinal perforation caused by bevacizumab for breast cancer, which is of special interest because gastrointestinal perforations caused by bevacizumab are very rare in breast cancer.

**Case presentation:**

We describe the case of 54-year-old Japanese woman. She was diagnosed as having inoperable breast cancer T2 N1 M1 (pleura, peritoneum), Stage IV, and received chemotherapy by paclitaxel. There was reduction in the primary tumor and disappearance of the pleural effusion; however, the ascites did not change. We performed diagnostic laparoscopy which revealed that her whole peritoneum was thickened, and her small intestine, colon, and her omentum were grouped and formed an omental cake. We submitted a part of her peritoneum to pathological examination and diagnosed the peritoneum dissemination of breast cancer. On the basis of these results, paclitaxel and bevacizumab combination chemotherapy was started, and a decrease in ascites was seen. However, a gastrointestinal perforation occurred on 26th day of second cycle of bevacizumab + paclitaxel, and we performed an emergency operation. In the operation, the omental cake was resolved, and we could search the full length of the gastrointestinal tract. Two small perforations of her small intestine were seen. We performed simple closures for perforations, and peritoneal lavage and drainage. She was in a state of septic shock, but it improved. It was thought that the small intestinal perforations were caused by the bevacizumab-additional chemotherapy which was very effective.

**Conclusions:**

We report a very rare and valuable case. This case suggests that the risk of gastrointestinal perforation must be considered in a case of bevacizumab administration, and it is necessary to determine carefully the patient administered bevacizumab, regardless of the type of cancer.

## Background

Bevacizumab is a molecular target drug used for colorectal cancer, non-small cell lung cancer, breast cancer, and malignant glioma. Gastrointestinal perforation is known as a serious adverse event, but, for breast cancer, there are no reports of gastrointestinal perforation in Japan and there are very few reports in the world [1–5].

In this case report we describe the case of woman with a small intestinal perforation during bevacizumab combination chemotherapy for inoperable breast cancer. In addition, we performed an operation and were able to save her life. This is the first report of this kind in the English literature.

## Case presentation

We describe the case of a 54-year-old Japanese woman.

### Present illness

**Fig. 1:**
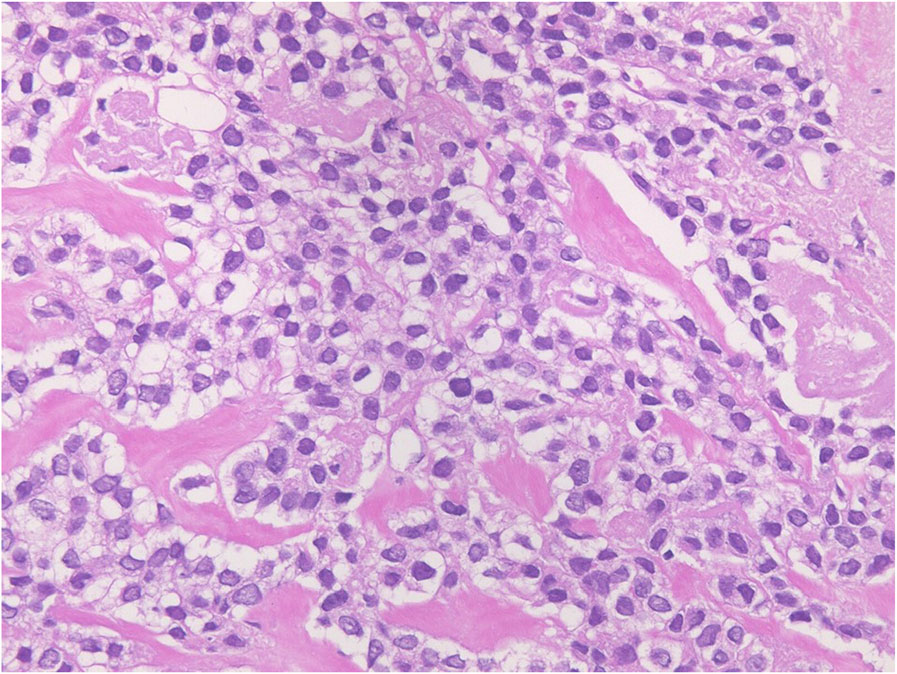
A solid increase in cells with clear cytoplasm were shown in core needle biopsy of the right breast tumor and a diagnosis of solid tubular carcinoma was made. Cytodiagnosis of pleural effusion and ascites showed similar cells

**Fig. 2:**
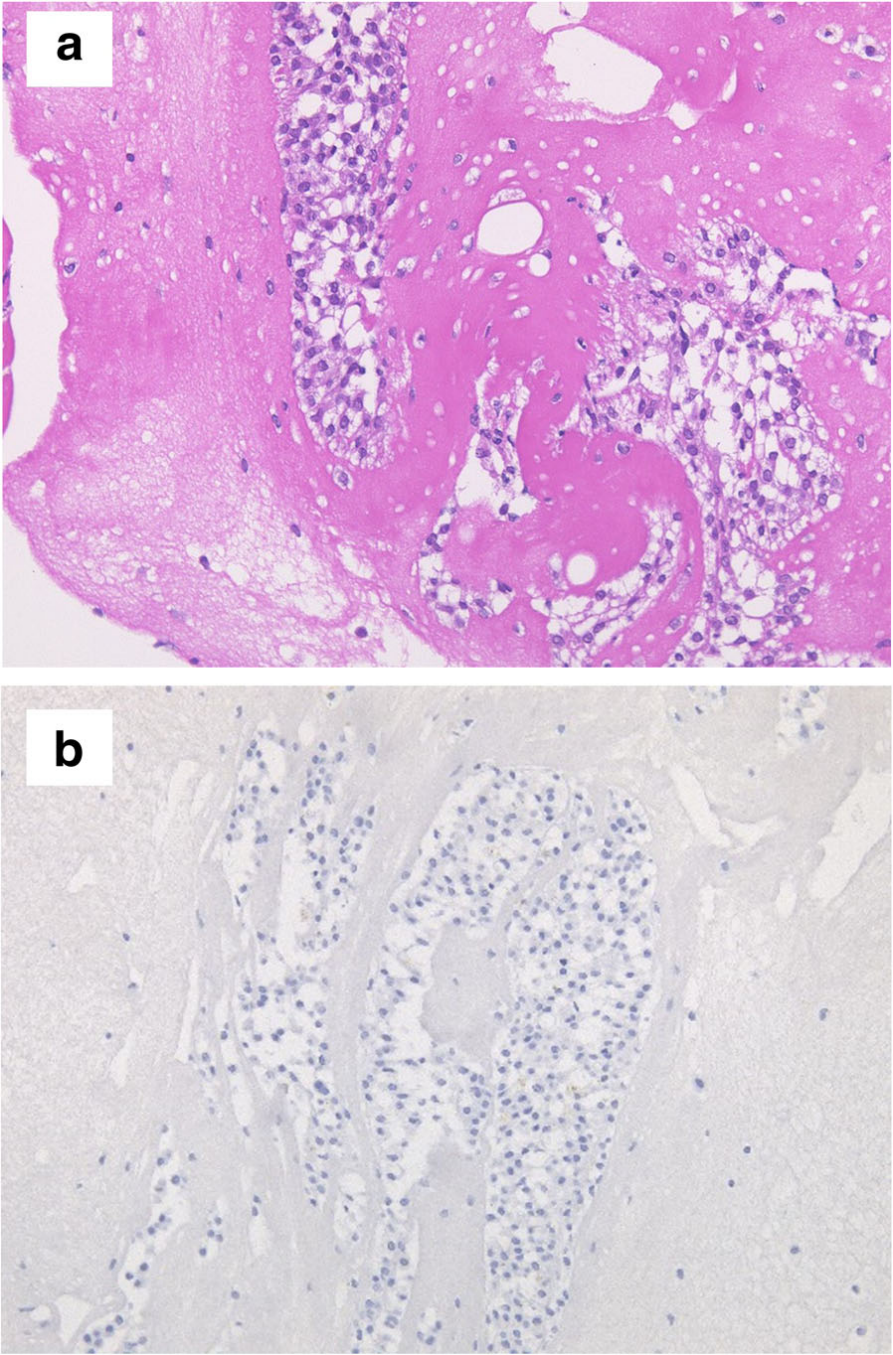
**a** The peritoneum dissemination of breast cancer was diagnosed by the pathological examination of part of the peritoneum. **b** Wilm's tumour tumor suppressor gene1 (WT1) staining of the peritoneum was negative

**Fig. 3:**
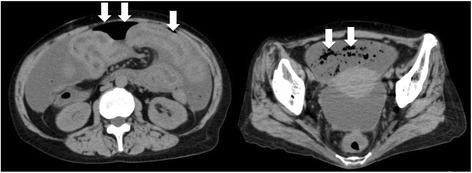
Ascites and abdominal free air (arrows) were shown by abdominal computed tomography

She went to a nearby clinic to get a check-up for abdominal distension. A large quantity of pleural and ascitic effusion was recognized and a blood test showed high CA125 (209 U/mL), and she was suspected to have a malignant tumor. She was admitted to a cancer center in our hospital, and we carried out a close examination. We found a tumor in her right breast and we performed a core needle biopsy. A diagnosis of primary breast cancer (solid tubular carcinoma) (Fig. 1) was made; estrogen receptor (EgR) and progesterone receptor (PgR) were positive, and human epidermal growth factor receptor 2 (HER2) was negative. Cytology of pleural and ascitic effusion revealed cells similar to the breast cancer, and she was diagnosed as having inoperable breast cancer, stage IV, according to TNM classification: T2 N1 M1, pleura, peritoneum. After these results, weekly paclitaxel therapy was started for our patient. The protocol of this therapy, as a cycle, was that paclitaxel (90 mg/m^2^ based on body surface area) was intravenously infused every week for 3 weeks followed by 1 week for rest. After three cycles of weekly paclitaxel, the primary tumor had reduced and subsequently the pleural effusion disappeared but the ascites did not change. Because only the ascites did not decrease, other cancers such as a peritoneal carcinoma were suspected and we performed diagnostic laparoscopy. A large amount of transparent and viscous ascites was detected in her abdominal cavity. Her small intestine, omentum, and transverse colon had become grouped into a dumpling form, and the whole peritoneum was thickened and hyperplasic. We excised a peritoneal sample and submitted it to histopathological examination. The histopathological examination revealed that the peritoneum was not the primary peritoneal cancer but the peritoneal dissemination of breast cancer because the peritoneal cells were similar to the cells of the primary breast cancer and there was no Wilm's tumour tumor suppressor gene1 (WT1) staining (Fig. 2). On the basis of these results, paclitaxel and bevacizumab combination chemotherapy was started as a second-line treatment and the ascites decreased. The protocol of this combination therapy, as a cycle, was that paclitaxel (90 mg/m^2^) was infused every week for 3 weeks followed by 1 week for rest. And bevacizumab (10 mg/m^2^) was infused following paclitaxel treatment on weeks 1 and 3 of every cycle. On the 26th day of the second cycle of this therapy, she had stomachache and abdominal computed tomography (CT) showed intraabdominal free air (Fig. 3); she was diagnosed as having a digestive perforation and an emergency operation was performed.

### Previous history

She underwent bilateral total hip replacement arthroplasty because of congenital hip dislocation 30 years ago. She was continuously medicated because of severe depression.

### Physical examination just before emergency operation

The state of septic shock was recognized with clear consciousness, high body temperature (38.5 °C), tachycardia (160 beats per minute), low blood pressure (61/35 mmHg), and high respiration rate (24 per minute). Her abdomen was tympanic and hard with whole abdominal tenderness and muscular defense.

### Laboratory data just before emergency operation

Her levels of leukocytes and C-reactive protein were elevated, and her renal function had degenerated. A blood gas analysis showed metabolic acidosis, vicarious respiratory alkalosis, and hyperlactacidemia (Table 1).

**Table 1:** Elevation of leukocytes and C-reactive protein, and degeneracy of renal function. A blood gas analysis showed metabolic acidosis, vicarious respiratory alkalosis, and hyperlactacidemia

Laboratory data		
Complete blood count		
WBC	8080	/μL
RBC	4.56	× 10^6^/μL
Hb	14.5	g/dL
Hct	40.7	%
Plt	18.7	× 10^4^//μL
Blood coagulation test		
PT	80.5	%
PT-INR	1.05	
APTT	29.0	seconds
Blood gas analysis		
pH	7.497	
PaO_2_	112.0	mmHg
PaCO_2_	16.5	mmHg
HCO^3^	12.7	mmol/L
BE	−10.2	mEq/L
Lactate	80.0	m/dL
Blood biochemical test		
TP	3.9	g/dL
Alb	1.6	g/dL
T-bil	0.2	mg/dL
AST	29	IU/L
ALT	14	IU/L
LDH	387	IU/L
CK	47	IU/L
BUN	27.4	mg/dL
Crea	1.01	mg/dL
Na	130	mmol/L
K	3.8	mmol/L
Cl	98	mmol/L
Ca	8.4	mg/dL
CRP	2.37	mg/dL

*Alb* albumin, *ALT* alanine aminotransferase, *APTT* activated partial thromboplastin time, *AST* aspartate aminotransferase, *BE* base excess, *BUN* blood urea nitrogen, *Ca* calcium, *CK* creatine kinase, *Cl* chlorine, *Crea* creatinine, *CRP* C-reactive protein, *Hb* hemoglobin, *HCO*_*3*_ bicarbonate, *Hct* hematocrit, *K* potassium, *LDH* lactate dehydrogenase, *Na* sodium, *PaCO*_*2*_ partial pressure of carbon dioxide in arterial blood, *PaO*_*2*_ partial pressure of oxygen in arterial blood, *Plt* platelets, *PT* prothrombin time, *PT-INR* prothrombin time-international normalized ratio, *RBC* red blood cells, *T-bil* total bilirubin, *TP* total protein, *WBC* white blood cells

### Abdominal computed tomography findings

Ascites and intraabdominal free air were shown.

### Operation findings

Abundant dirty ascites was detected. The omental cake was resolved, and we were able to search the full length of the gastrointestinal tract. At the time of diagnostic laparoscopy, her small intestine, her omentum, and her transverse colon formed part of the omental cake, but the adhesion was separated in this emergency operation and all intestinal searches were enabled. Thus, it was considered that digestive perforation was caused by the strong efficacy of bevacizumab combination chemotherapy. Two small perforations of her small intestine were seen; they were located at a distance of 120 cm from the Treitz ligament. We performed simple closures of perforations, and peritoneal lavage and drainage.

### Course after emergency operation

Before the emergency operation, septic shock was recognized, and after the operation, in addition, she developed disseminated intravascular coagulation and oliguria due to acute renal failure. We used continuous venous injection of noradrenaline and vasopressin together, and under artificial breathing management in our intensive care unit she gradually improved. She was removed from the respirator 3 days after the operation and gradually weaned from the vasopressor until it was discontinued. However, her nutritional status progressed to bad, and her level of activity decreased from crutch walking until she became approximately bedridden. Depression contributed to a decrease in our patient’s will for treatment. These were judged generally and on enough informed consent she received anticancer therapy of only orally administered aromatase inhibitor and died from the original breast cancer 9 months after surgery.

## Discussion

Vascular endothelial growth factor (VEGF) is expressed in many carcinomas; furthermore, it is known to be associated with invasion, metastasis of tumor, recurrence, and prognosis. Bevacizumab is a chimeric hominization immunoglobulin G1 monoclonal antibody directed against VEGF. Bevacizumab inhibits the neovascularization (formation and growth) in the tumor tissue and can delay tumor growth by inhibiting binding with a receptor developing in VEGF and vascular endothelial cells. The effect of not only neovascularization antagonism but also the effects to reduce tissue framework pressure of the tumor by making the vasculature of the tumor normalize and to improve antineoplastic accessibility to a tumor, was suggested [6, 7]. High efficacy is shown by combination with antineoplastic and other molecular target drugs in various kinds of types of cancer. For breast cancer, bevacizumab with paclitaxel combined chemotherapy was authorized in Japan in September 2011.

While efficacy of bevacizumab is reported, characteristic and serious adverse events are being reported, including hypertension, proteinuria, hemorrhage, arterial thrombosis, protraction of wound healing, fistula formation, and gastrointestinal perforation [8–12]. The potential of gastrointestinal perforation was reported for 0.9% of patients treated with bevacizumab, and the fatality rate of patients with gastrointestinal perforation treated with bevacizumab was reported to be as high as 20% [8, 12]. Of gastrointestinal perforations induced by bevacizumab, 80% occur within 6 months after initial bevacizumab administration [8]. The most common site of perforation is the colon, while small intestine ranked second and stomach ranked third [8]. The risk factor of gastrointestinal perforation includes a high-dose administration of bevacizumab, a history of abdominal radiotherapy, carcinomatous peritonitis, colorectal cancer, renal cell carcinoma, colonic diverticulitis, peptic ulcer, and administration of steroids or nonsteroidal anti-inflammatory drugs (NSAIDs) [8]. It is considered that causes of bevacizumab-related gastrointestinal perforation are disorder of the structure and function of blood vessels, protraction of wound healing, and failure of tumor. Several mechanisms had been described to explain the development of gastrointestinal perforation as a result of bevacizumab. In the first, the inhibition of VEGF by bevacizumab could cause a decrease in vascular density, a disorder of vasoconstriction due to the release disorder of nitric monoxide, arterial thrombus, embolism as a result of failure of blood vessel structure, and disorder of functions [6–13]. The second possible mechanism is related to gastrointestinal mucosa. Constant gastrointestinal wall proliferation and healing is dependent on microcirculation, protection with nitrous oxide, and normal platelet function, all of these depend on VEGF [6–13]. Intestinal healing after damage such as surgery or ulcers is also dependent on these processes. From these mechanisms, NSAIDs are a risk factor of bevacizumab-related gastrointestinal perforation. The third possible mechanism is, ironically, the great efficacy of bevacizumab to reduce tumor volume [6–13]. Tumor structure may provide some stability to the intestinal wall itself, and tumor death caused by bevacizumab creates perforation. There is no consensus for the pathological findings because gastrointestinal perforation cases caused by bevacizumab are rare and they could be caused by many mechanisms (as previously mentioned). A search using the terms “breast cancer,” “bevacizumab,” and “perforation” in PubMed for the period 1950 through to 2015, retrieved two case reports from France and 12 cases in clinical trials of bevacizumab: E2100, RIBBON-1, RIBBON-2, and AVADO. In total, only 14 cases were reported [1–5]. The two cases in France were small intestinal perforation without intraabdominal cancer lesion [1, 2].

In our case, when we performed diagnostic laparoscopy, all the intestinal tract and omentum formed an omental cake, and the whole peritoneum was thickened. A histopathological diagnosis of peritoneal dissemination of the breast cancer was made from a peritoneal sample. On the basis of these results, bevacizumab combined therapy was started; however, a small intestinal perforation occurred approximately 2 months after the initial bevacizumab administration. Therefore, an emergency operation was performed. In consideration of the intraoperative findings and onset time, bevacizumab was very effective and it was considered that the small intestinal perforation was caused by the failure of the tumor. For the gastrointestinal perforation caused by bevacizumab, it is safe that construction of enterostomy using oral tract from the perforation in consideration of protraction of wound healing [14]. However, only a few cases in which the perforated tract was excised and anastomosis was performed at the same time and simple closure was performed, were reported [15]. In our case, the perforations were 120 cm distant from Treitz ligament and located in upper small intestine, and serosa near perforations was considered almost normal, not edematous or inflammatory. Therefore, in consideration of worsening of quality of life caused by enterostomy, we performed a procedure of simple closure; however, in consideration of that she received treatment to elevate her blood pressure by peripheral vasoconstriction because she was critically ill with septic shock, in addition to protraction of wound healing by bevacizumab, the possibility of anastomotic leakage was enough. Therefore, we checked the drain finely and were able to re-operation at anytime, but, anastomotic leakage was not caused fortunately. Evading enterostomy with upper small intestine was considered a very big benefit to our patient. However, after all we should be very careful about a simple closure or resection and single-stage anastomosis. Scrupulous postoperative follow-up is necessary, including tight checking the drain, to ensure that a reoperation is performed immediately in the case of an emergency.

## Conclusion

In the case of a patient receiving bevacizumab administration, the risk of gastrointestinal perforation should be considered, and regardless of the type of cancer, careful administration bevacizumab is necessary.
